# Octreotide-Treated Diabetes Accompanied by Endogenous Hyperinsulinemic Hypoglycemia and Protein-Losing Gastroenteropathy

**DOI:** 10.1155/2011/381203

**Published:** 2011-08-04

**Authors:** Nobuhiko Takahashi, Miho Nagamine, Mitsuko Fukuda, Wataru Motomura, Atsuko Abiko, Masakazu Haneda, Mikihiro Fujiya, Masahiro Ieko, Yutaka Kohgo

**Affiliations:** ^1^Department of Internal Medicine, School of Dentistry, Health Sciences University of Hokkaido, Ishikari-Toubetsu, Hokkaido 061-0293, Japan; ^2^Division of Gastroenterology and Hematology/Oncology, Department of Medicine, Asahikawa Medical University, Asahikawa, Hokkaido 078-8510, Japan; ^3^Division of Metabolism and Biosystemic Science, Department of Medicine, Asahikawa Medical University, Asahikawa, Hokkaido 078-8510, Japan

## Abstract

Occurrence of hypoglycemia in diabetes patients is very rare. We report here a case of frequent hypoglycemic attacks caused by inappropriate endogenous hyperinsulinemia in a female patient with poorly controlled diabetes and protein-losing gastroenteropathy. The blood glucose profiles of the patient were unstable. Results of the fasting test performed to investigate the cause of hypoglycemia suggested endogenous hyperinsulinism. Repeated selective arterial calcium injection tests suggested that hyperinsulinemia might be extrapancreatic in origin. However, efforts to detect a responsible lesion such as insulinoma were unsuccessful. Octreotide was used for the treatment of hypoglycemia and protein-losing gastroenteropathy. After treatment, although her leg edema caused by hypoalbuminemia persisted, hypoglycemia almost disappeared.

## 1. Introduction

Hypoglycemia is a clinical syndrome with diverse causes characterized by low levels of plasma glucose [1]. When it occurs in a patient with known diabetes, the blood glucose profiles are unstable or sometimes ameliorated [2]. To evaluate such hypoglycemia, one should consider many etiologies such as increased insulin sensitivity, increased insulin bioavailability, inadequate carbohydrate intake, mismatch of insulin and food intake, decreased clearance of insulin/oral agent, hypothyroidism, impaired counterregulation, and effect of some drugs [3]. Further, related serum tests such as those for fasting blood glucose, immunoreactive insulin, and insulin autoantibody were performed.

Endogenous hyperinsulinemic hypoglycemia such as insulinoma or nesidioblastosis is one of important causes of hypoglycemia and is suggested by a positive fasting test. To diagnose insulinoma, computed tomography (CT) scan, magnetic resonance imaging (MRI) scan, and endoscopic ultrasound were performed to localize the possible lesion. However, the diagnostic values of these tests are not high if the underlying insulinoma is a very tiny tumor or may be of extrapancreatic origin. Alternatively, if nesidioblastosis is the cause of hypoglycemia, the lesion is difficult to be depicted because it is characterized by diffuse or disseminated proliferation of islet cells arising from pancreatic ducts or ductules and is generally diagnosed only by pathology [4, 5]. To diagnose endogenous hyperinsulinemia functionally and anatomically, a selective arterial calcium injection (SACI) test is very useful [6].

Here, we report an extremely rare case of endogenous hyperinsulinemic hypoglycemia without distinct mass in a patient with poorly controlled diabetes and protein-losing gastroenteropathy. Moreover, we discuss the unusual clinical presentation, especially relating to the results obtained with SACI tests, the pathophysiology, and effective management of such cases.

## 2. Case Presentation

A 52-year-old female with a history of admission to the hospital 5 times in the last 5 years because of frequent hypoglycemic attacks was admitted to our hospital.

At the age of 36, her urine examination showed excessive glucose levels but she did not receive further medical treatment. The following year, she consulted a doctor due to general malaise and bilateral lower leg edema. She was then diagnosed with diabetes mellitus and hypoalbuminemia of unknown cause. Glibenclamide was administered for the treatment of diabetes at age of 40, but poor control persisted. She was referred to our hospital for the first time at the age of 43 because of exacerbation of her lower leg edema. Protein-losing gastroenteropathy (PLGE) due to idiopathic gastrointestinal lymphangiectasia was diagnosed by functional and histopathological examinations. She had been treated with elemental diet and steroid therapy for her PLGE, but those treatments were not effective. At the same time, insulin therapy was started because of the poorly controlled diabetes (HbA1c, approximately 10%). After the age of 47, her average blood glucose levels were keeping consistently high (300 to 400 mg/dL) and even hypoglycemic attacks had been recognized. When she was hospitalized at the age of 48, the cause of the hypoglycemic attacks was unidentified (2004). Around the age of 50, the frequency of hypoglycemic attacks (defined as blood glucose level less than 70 mg/dL) had increased to about 40 times per month. The attacks occurred at postprandial and preprandial times. Her medication history suggested that hypoglycemia was not related to her insulin therapy. Precise examination of hypoglycemia was performed during the current hospitalization at the age of 52.

**Figure 1:**
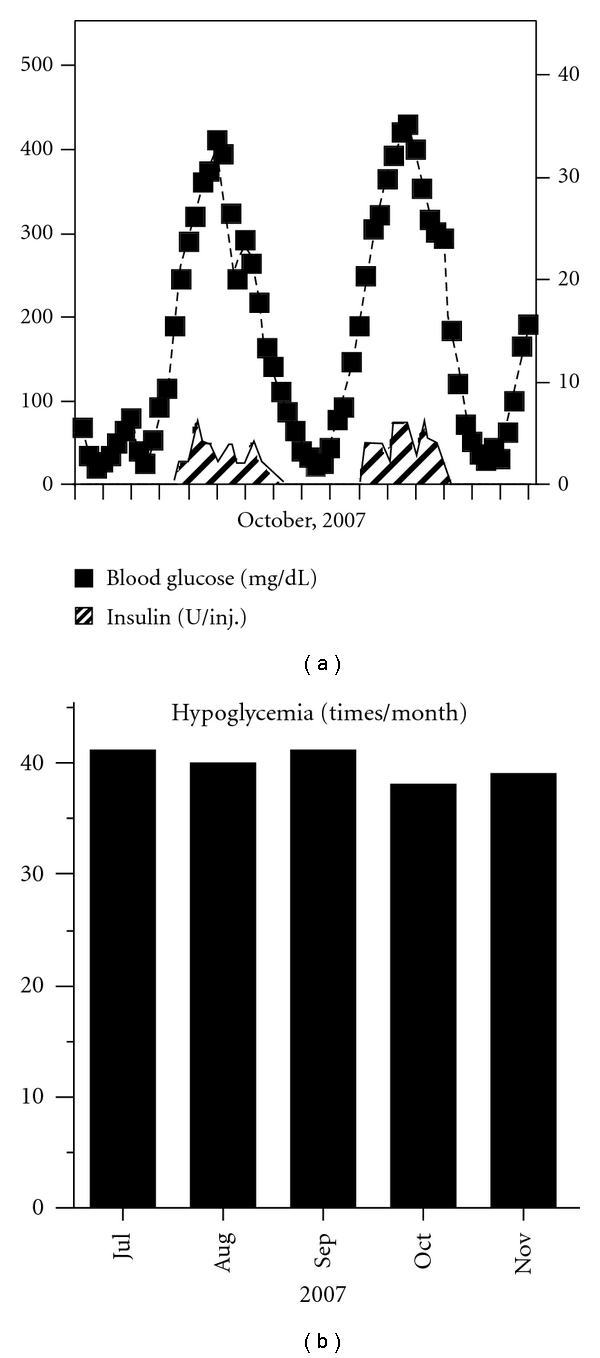
Representative blood glucose profiles before treatment. Blood glucose profile and the dose of each insulin injection are shown (a). The duration between ticks on the *X*-axis represents 24 h. The frequency of hypoglycemia, defined as below 70 mg/dL of blood glucose, is shown (b).

On admission, her body height was 137 cm and body weight was 44 kg (BMI, 23.4 kg/m^2^). Blood pressure was 128/78 mmHg and heart rate was 87. Physical examination revealed bilateral lower leg pitting edema, no Achilles tendon reflexes, and reduced sensation of vibration (C128 Hz, 4 s (rt.)/4 s (lt.)). Chest X-ray revealed no pleural effusions and no cardiomegaly. Diabetic complications were confirmed, that is, bilateral proliferative retinopathy, nephropathy (stage 2), and peripheral neuropathy. She was treated with a combination of rapid-acting and NPH insulin injection for her diabetes. The results of laboratory tests were as follows: total protein: 4.0 g/dL; serum albumin: 2.3 g/dL; total cholesterol: 172 mg/dL; triglyceride: 104 mg/dL; HDL-c: 47.4 mg/dL; LDL-c: 116.7 mg/dL; creatinine: 0.49 mg/dL; HbA1c: 15.5%; urinary C-peptide: 125 *μ*g/day; anti-GAD and anti-IA-2 antibodies: negative; urinary microalbumin: 73.8 mg/g·Cre; creatinine clearance: 109.4 mL/min/m^2^. Blood glucose profiles appeared unstable, with periodic (2 to 3 days) alternation between hyperglycemia (>300 mg/dL) and hypoglycemia (<70 mg/dL). Representative blood glucose profiles are shown in Figure 1(a), and the frequency of hypoglycemia is shown in Figure 1(b). A glucagon loading/stimulation test revealed that the ability of insulin secretion was retained (ΔCPR = 4.8 ng/mL). No abnormalities were found in other endocrine hormones.

**Figure 2:**
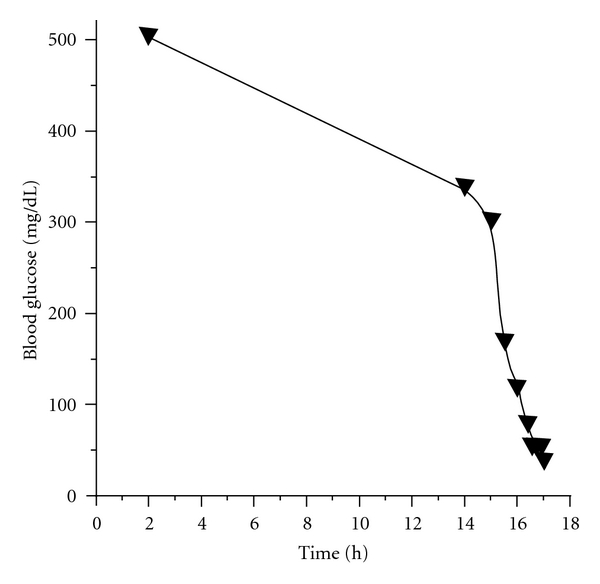
The result of the fasting test. Blood glucose levels were monitored. After 17 h of fasting, the blood glucose level decreased to 45 mg/dL.

Because the hypoglycemic attacks occurred at a time after cessation of insulin injections sufficiently longer than the effective time of the insulin, we were convinced that hypoglycemia was not related to the injections. Therefore, we suspected that it was induced by an endogenous cause. The result of a fasting test was positive (Figure 2). After a 17 h fast, hypoglycemia (plasma glucose 45 mg/dL) appeared and serum insulin concentration was 9.92 *μ*U/mL (normal, <6.0), C-peptide level was 0.98 ng/mL (<0.6), and proinsulin level was 15.3 pmol/L (<5.0). Service's and Turner's criteria for diagnosing insulinoma were satisfied (Grunt 4.54 (positive, <2.5), Fajans 0.22 (>0.3), Turner 66.1 (>50), and Service 9.92 (>6)). Serum C-peptide and proinsulin levels at the time of hypoglycemia attack were significantly high, and the values satisfied the criteria for hyperinsulinism defined by Service [1]. Also, the proinsulin level was above 5 pmol/L, and this parameter and threshold level have been reported as the best criteria for diagnosis of endogenous hyperinsulinism during the fasting test [7]. Counterregulatory response to hypoglycemia was not impaired; before and at the time of hypoglycemia, testing showed the following respective levels: glucagon 150 pg/mL and 230 pg/mL; cortisol 6.09 *μ*g/dL and 27.45 *μ*g/dL; growth hormone 0.33 ng/mL and 11.70 ng/mL; adrenaline <10 pg/mL and 583 pg/mL. Tests for both anti-insulin antibody and anti-insulin receptor antibody were negative. We excluded the specific hypoglycemic conditions induced by medicines, insulin autoimmunity, factitious hypoglycemia, and other conditions/diseases such as renal failure or liver cirrhosis. We did not measure plasma sulfonylurea concentration as we were aware that the patient had not been taking the medicine. To investigate the possible involvement of glucose-dependent insulinotropic polypeptide (GIP) or glucagon-like peptide 1 (GLP-1) in the hypoglycemia, we measured serum concentrations of total GIP (Human GIP Assay Kit, Immuno-Biological Laboratories, Fujioka, Japan) and total GLP-1 (YK160 GLP-1 EIA Kit, Yanaihara Institute, Fujinomiya, Japan) by enzyme-linked immunosorbent assays in her fasting, postprandial, and hypoglycemic state. Serum concentrations of total GIP were 7.6, 30.2, and 6.0 pg/mL, whereas those of total GLP-1 were 4.7, 4.2, and 4.1 ng/mL in fasting, postprandial, and hypoglycemic state, respectively. Taken together, these results suggested that inappropriate endogenous hyperinsulinemia was responsible for her hypoglycemia, possibly due to insulinoma, nesidioblastosis, or *β* cell dysfunction.

**Figure 3:**
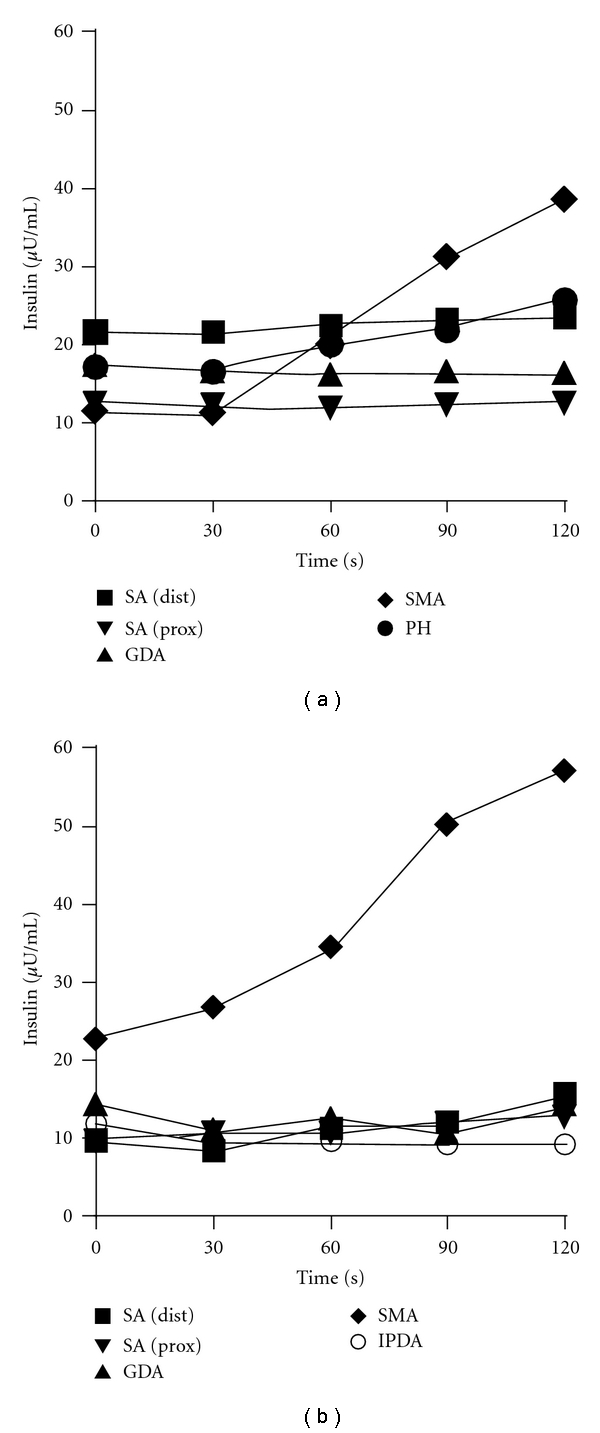
Results of SACI tests at 2004 (a) and 2008 (b). A vascular catheter was located to each specific artery (■ splenic (distal), ▲ splenic (proximal), ▾ gastroduodenal, ♦ supramesenteric, • proper hepatic, ∘ inferior pancreaticoduodenal artery). Blood samples were collected from the hepatic vein for measuring insulin concentrations before and after stimulation by calcium gluconate (Ca^2+^ 0.025 mEq/Kg).

Thus far, all efforts for obtaining diagnostic imaging of an insulinoma via CT scan, MRI scan, and endoscopic ultrasound were unsuccessful. SACI tests with calcium gluconate (Ca^2+^ 0.025 mEq/kg) were performed twice in 2004 (age 48) and 2008 (age 52). In keeping with other researchers, an increment in plasma immunoreactive insulin levels greater than 2-fold over prestimulation levels was considered indicative of a tumor or other responsive lesion in the vascular territory of the artery studied [8]. The results, confirmed twice, were positive only after stimulations of the supramesenteric artery (SMA) (2.53-fold increase in 2004 and 3.36 in 2008 versus before stimulation; Figures 3(a) and 3(b), resp.). No anatomical variant was apparent on review of the angiogram, but there seemed to be backflow slightly in the gastroduodenal artery (GDA) on the examination only in 2008. Exploratory laparotomy was refused by the patient and her family.

**Figure 4:**
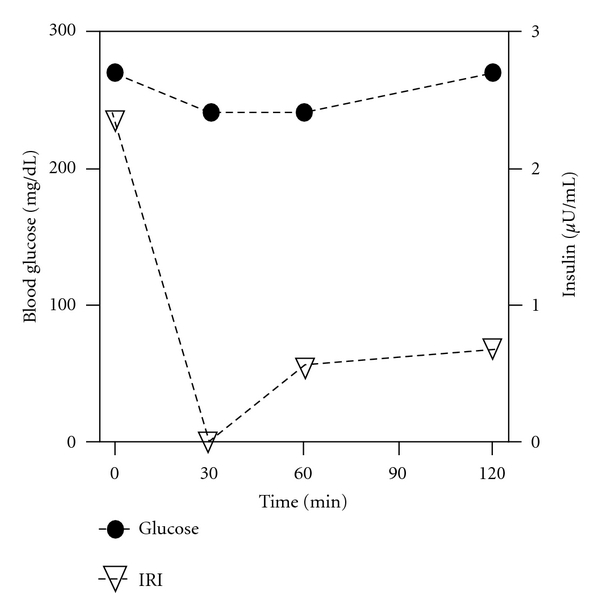
The effects of a single dose of octreotide (50 *μ*g) on blood glucose and serum immunoreactive insulin levels.

**Figure 5:**
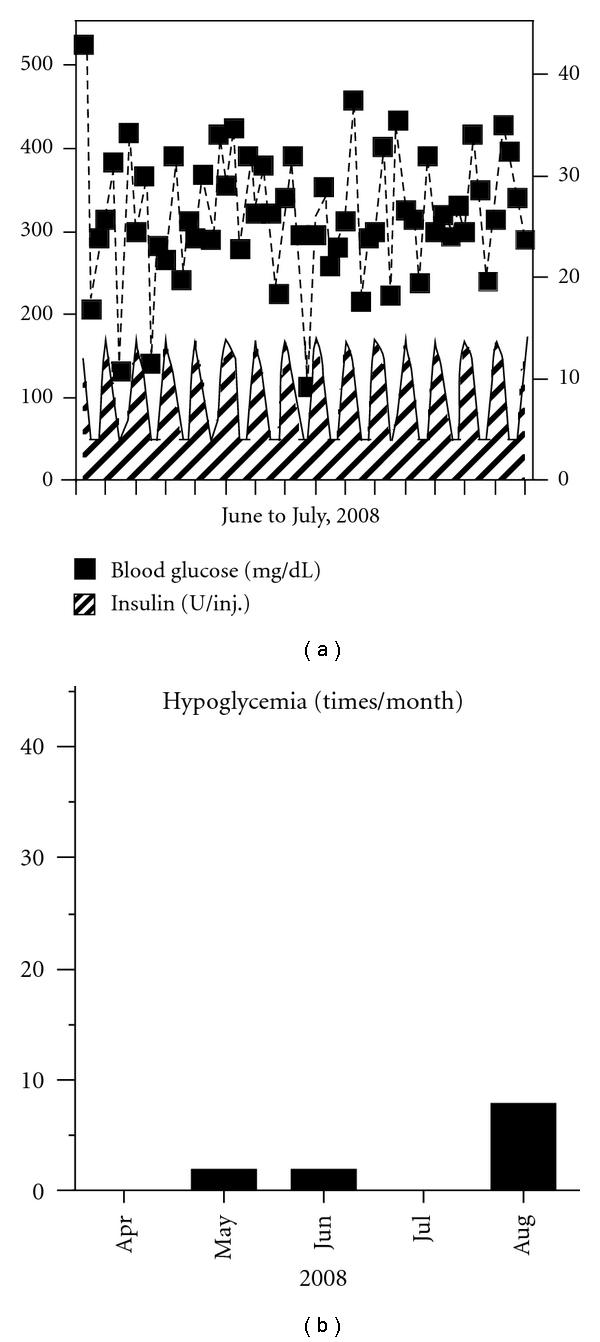
Representative blood glucose profiles after the treatment of octreotide LAR for 4 months. Blood glucose profile and the dose of each insulin injection are shown (a). The duration between ticks on the *X*-axis represents 24 h. The frequency of hypoglycemia, defined as below 70 mg/dL of blood glucose, is shown (b).

Since she had been suffering mostly from frequent hypoglycemic attacks, we focused primarily on the management of hypoglycemia by medication. We considered the use of octreotide (Sandostatin, Novartis, Basel, Switzerland), a long-acting somatostatin analog, because this medicine suppresses insulin secretion [9] and has been reported to be effective for PLGE due to idiopathic gastrointestinal lymphangiectasia [10]. At first, we tried a single dose (50 *μ*g) of octreotide to evaluate its effects on serum insulin and glucose levels (Figure 4). As we observed no remarkable changes in glucose levels and suppression of insulin levels, we started her on octreotide (50 *μ*g, twice a day) for 2 weeks, monitoring its tolerability. Since almost no side effects except for controllable constipation were determined, we switched to octreotide LAR (Sandostatin LAR, Novartis, 20 mg per month), which is a very long-acting octreotide. After using octreotide LAR for 4 months, although hyperglycemia was uncontrollable even with multiple insulin injections (Figure 5(a)), the frequency of hypoglycemic attacks was surprisingly reduced (Figure 5(b)). This effect was sustained for 20 months until her death from acute heart failure (54 years, 2010). On the other hand, serum albumin was slightly increased (2.3 g/dL and 3.0 g/dL; before and after treatment, resp.), but her leg edema was unchanged.

## 3. Discussion

To the best of our knowledge, this is the first report of an extremely rare case with the following four characteristics: (1) frequent hypoglycemia induced by endogenous hyperinsulinemia derived from an unusual origin without distinct mass, (2) hypoglycemia in a patient with known diabetes, (3) coexistence with PLGE, and (4) amelioration of hypoglycemia by treatment with octreotide.

The most interesting and important point in this case is that positive results from both SACI tests were obtained only via SMA stimulation. The SACI test is an important and useful method for localization of the lesion responsible for hyperinsulinism such as insulinoma. A 2-fold or greater stepup in hepatic vein insulin concentration from baseline after arterial calcium injection constitutes a positive response [8]. Using this criteria, Guettier et al. reported that a positive response was observed in a single vessel in 16 of 45 cases (36%) and in two or more vessels in 25 of 45 cases (53%) [11]. The reasons for this are unclear and may relate to overlap in arterial territory, tumor behavior, or problems related to the specificity of the test. When multiple arterial injections elicit a positive response, the dominant response is taken as the site of tumor localization. The sensitivity of SACI in localizing insulinomas has ranged from 88% to 94% [8, 12, 13], and a false positive rate is reported to be 4% [11]. We performed SACI twice in our case when she was 48 and 52 years old, and positive results were obtained reproducibly in both tests for stimulations performed only via SMA. We believe this was reliable evidence for SMA localization. In terms of vascular territories for the pancreas, SMA supplies the uncinate process and secondarily the pancreatic head in addition to the mesentery, and GDA supplies the pancreatic head and secondarily the uncinate process. If a lesion existed within the pancreas in this patient, the positive results via SMA stimulation suggest that the lesion would have been located in the head/uncus of the pancreas. If a lesion was located in that part of the pancreas, a positive result could also have been obtained from GDA stimulation. However, we failed to confirm positive GDA stimulation twice. Because a backflow into GDA was observed by angiography at the age of 52, the stimulation via GDA might have been insufficient. Therefore, an additional and selective injection into the inferior pancreaticoduodenal artery (IPDA), the branching artery of SMA leading to the pancreatic head, was performed to investigate a possible lesion in the head/uncus of the pancreas [14, 15]. Consequently, stimulation via IPDA in this case was negative on the second SACI test. Taken together, we concluded that hypoglycemia was induced by endogenous hyperinsulinism and the responsible lesion possibly existed in the vascular territory of SMA that was of extrapancreatic origin (including the surroundings of the pancreas).

The delayed maximum reaction time (120 s) observed in both SACI tests was characteristic. In general, the maximum reaction time in the SACI test is observed 30–60 s after calcium injection. It has been reported that malignant insulinomas showed a slow increase with a peak observed 90–120 s after stimulation, depending on size and neovascularization [16]. However, there were no signs or suggestions from imaging of a malignancy in our case. A few cases of benign insulinoma showing a delayed response in the SACI test have been reported [17, 18]. Tsagarakis et al. reported a case of insulinoma located in the junction between the neck and body of the pancreas, and the SACI test of the patient showed a maximum rise at 180 s after stimulation from GDA [17]. O'Shea et al. reported a similar case of insulinoma in the pancreatic tail, and the SACI test showed the maximum rise at 120 s after stimulation from the splenic artery [18]. The reason why the maximum reaction was delayed in these cases has not been clarified; however, it may have been due to slow and/or complicated blood flow or altered *β* cell tumor responses. Taking the results of these reports into account, the delayed response observed in our case should be considered as significant.

The differential diagnosis of endogenous hyperinsulinemic hypoglycemia in this patient listed extrapancreatic insulinoma and nesidioblastosis. Filipi and Higgins reported that the frequency of ectopic insulinoma was 1.96% (20/1018) including the wall of the duodenum (5/20), near the tail of the pancreas (3/20), near the pancreas (5/20), gastrosplenic omentum (2/20), the paraduodenal region (2/20), and between the head of the pancreas and liver (1/20) [19]. Another report showed that the frequency of ectopic islet tumor was 2.26% (9/398) [20]. According to the SACI results in this case including positive SMA and negative GDA and IPDA stimulations, an undetectable ectopic insulinoma may have been present in the vascular territory of SMA. Nesidioblastosis has traditionally been thought to be the most common cause of endogenous hyperinsulinism in neonates and infants [4]. By comparing clinical features with insulinoma, hypoglycemia arising from nesidioblastosis tends to occur at postmeal times, as partly seen in our case. Considering these points, it may be possible that a nesidioblastic change occurred in the ectopic pancreas of the patient. Alternatively, nesidioblastosis might have existed in the head of the pancreas and not been detected if the GDA and IPDA stimulations to the patient were insufficient.

The development of endogenous hyperinsulinemic hypoglycemia in patients with known diabetes is extremely rare. Most of these cases have been due to benign insulinomas. In addition, islet hyperplasia and nesidioblastosis were reported in hypoglycemic patients with diabetes [21]. The blood glucose profiles for diabetes accompanied by insulinoma are unstable, similar to those in our case as shown in Figure 1(a). The similarity of the profiles convinced us that our patient also had either insulinoma or nesidioblastosis. It is difficult to know the pathogenic relationship between her existing diabetes and hyperinsulinemia. Chronic insulin exposure alters insulin signaling in vitro. For example, protein levels of insulin receptor substrate-1, which play important roles in insulin signaling, are reduced in adipocytes [22]. Insulin resistance is shown in insulinoma patients as compared with healthy nonobese controls by hyperinsulinemic clamp studies, in addition to obese type 2 diabetic patients [23]. For our case, these results suggested that altered insulin sensitivity induced by hyperinsulinemia may have partially contributed to her unstable blood glucose control.

The relationship between hyperinsulinemia and gut diseases including PLGE and intestinal lymphangiectasia remains unclear. Gut secretes incretins such as GIP and GLP-1, and both peptides stimulate insulin secretion from pancreatic *β* cells [24]. In Caucasian subjects, serum concentrations of these peptides are increased in response to meal ingestion compared with fasting state [25, 26]. Additionally, increased GLP-1 concentration has been implicated in reactive hypoglycemia in patients with gastrectomy or gastric bypass surgery [27, 28]. Given the insulinotropic properties of incretins, we hypothesized that GIP or GLP-1 might be involved in her hypoglycemia. As shown in Section 2, we observed a rise in GIP but not in GLP-1 concentration in postprandial state. Interestingly, a recent study in Japanese subjects has demonstrated that no obvious peak of total GLP-1 was seen after meal ingestion [29]. The racial difference in incretin system may account for the loss of GLP-1 response after meal ingestion. No significant changes were seen in the concentrations of both peptides in hypoglycemic state compared with those in fasting state. Although our measurements were limited in number, these results suggest little possibility of the involvement of incretins in her hypoglycemia.

We considered a medication for appropriately raising her blood glucose levels and thus alleviating the symptom of hypoglycemia. The possible medicines listed were glucocorticoid, glucagon, diazoxide, somatostatin analog, and streptozotocin. Previously, we administered prednisolone for the treatment of PLGE but the treatment was not effective. Treatment of glucagon is difficult to continue for a patient because it needs to be administered intramuscularly or intravenously. Streptozotocin is an approved medicine for treatment of malignant islet cell tumor by destructing islet cells. Because the effect of the drug is irreversible to islet cells, thereby exacerbating diabetes inevitably, we could not select this medicine. Diazoxide was originally developed as an antihypertensive medicine but later found to inhibit insulin secretion by activating the potassium channel in *β* cells. However, approximately half of the patients administered diazoxide suffered from several adverse effects including edema [30]. We worried about exacerbation of her existing edema caused by PLGE with diazoxide treatment. In addition, it was not available commercially in Japan at the time of treatment for this patient. Novel diazoxide analogs that have potent and selective activation of the pancreatic *β* cell type K_ATP_ channel have been developed recently [31], and these medicines will be used for an alternative treatment in future.

Octreotide, a somatostatin analog, is effective against endogenous hyperinsulinemic hypoglycemia by inhibiting insulin secretion [9]. Furthermore, this medicine has also been used for the treatment of PLGE [10]. Because of these properties, we gave preference to the use of octreotide. Vezzosi et al. recently reported that octreotide was effective against hyperinsulinemic hypoglycemia in 14 of 21 patients [9]. Octreotide was also effective in raising blood glucose in hypoglycemia caused by sulfonylurea [32]. On the other hand, octreotide improved blood glucose control in combination with insulin in diabetes by inhibiting glucagon secretion and intestinal absorption [33]. The addition of octreotide to insulin therapy in diabetic patients has been reported to give improved glycemic control despite reduction of insulin dose [34]. Therefore, since octreotide suppresses both insulin and glucagon secretion, it could not be predicted whether blood glucose levels would increase or decrease with this medicine for this patient. Therefore, we first confirmed its tolerability in the patient by a single-dose test and subsequent 2-week administration. After treatment with octreotide, the frequency of hypoglycemic attacks was dramatically reduced and her quality of life was greatly improved, although her blood glucose remained high. Octreotide increased her serum albumin slightly, but her leg edema was unchanged.

Regarding the side effects of long-term use of octreotide, Vezzosi et al. reported that only one patient had a symptomatic biliary lithiasis that appeared to be caused by the medicine among 11 patients who received treatment with the medicine over 6 months [9]. During the 20-month treatment in our case, we recognized no apparent side effects. Treatment with the somatostatin analog has been shown to shrink the size of some endocrine tumors [35, 36]. However, there have been no reports on such an effect of the drug on insulinoma to date. When using octreotide, careful monitoring is needed, and one needs to weigh the benefits against the side effects.

To our knowledge, this is the first report of a diabetic patient with PLGE diagnosed with endogenous hyperinsulinemic hypoglycemia without distinct mass. We managed frequent hypoglycemic attacks with octreotide effectively.

## Abbreviations

PLGE: Protein-losing gastroenteropathy
SACI: Selective arterial calcium injection
SMA: Supramesenteric artery
IPDA: Inferior pancreaticoduodenal artery.
